# Covariance-based analysis of spindle-band EEG during declarative and non-declarative odor cueing in sleep

**DOI:** 10.3389/fnins.2026.1810323

**Published:** 2026-06-18

**Authors:** Jesyin Lai, Pankaj Pandey, David M. Baum, Jens G. Klinzing, Andrea Sánchez-Corzo, Ranganatha Sitaram

**Affiliations:** 1 Department of Radiology, St. Jude Children’s Research Hospital, Memphis, TN, United States; 2 InteraXon Inc. (Muse), Toronto, ON, Canada; 3 Institute of Medical Psychology and Behavioral Neurobiology, University of Tübingen, Tübingen, Germany; 4 Centre for Integrative Neuroscience, University of Tübingen, Tübingen, Germany; 5 Department of Psychology, Ludwig Maximilian University of Munich, Munich, Germany

**Keywords:** covariance-based decoding, explainable machine learning, memory consolidation, Riemannian Geometry, sleep EEG, spindle-band oscillations, targeted memory reactivation (TMR)

## Abstract

**Introduction:**

Sleep supports memory consolidation through the reactivation of neural circuits engaged during learning. Targeted Memory Reactivation (TMR), in which memory-associated sensory cues are presented during sleep, can enhance declarative memory retention. However, the neural signatures supporting odor-cued reactivation remain incompletely characterized.

**Materials and methods:**

Here, we analyzed high-density electroencephalography (EEG) recordings from a TMR paradigm designed to dissociate neural responses associated with declarative and non-declarative odor cueing during sleep. EEG epochs were examined across fast- (12.5–16 Hz) and slow-spindle (9–12.5 Hz) frequency bands, channel subsets (all, frontal, central, and posterior), and multiple post-cue time windows (0–2, 0–4, and 0–7 s). Using within-participant machine learning based on Riemannian geometry, we classified EEG epochs elicited by a declarative memory-associated odor (Odor D) vs. vehicle control, and by a non-declarative odor associated with a motor task (Odor M) vs. vehicle.

**Results:**

Decoding performance relative to permutation-derived chance showed condition-dependent patterns across frequency bands, time windows, and channel subsets. Across analyses, decoding tended to be higher in the declarative condition (Odor D) than in the non-declarative condition (Odor M), with the strongest effects observed in central channels. Channel-level contribution analysis further indicated more spatially structured covariance patterns during Odor D over central regions, whereas contributions during Odor M were more diffuse and less consistent. These effects were modest and did not survive correction for multiple comparisons.

**Discussion:**

These results suggest that declarative odor cueing during sleep is associated with more structured spindle-band EEG patterns than non-declarative cueing, particularly over central channels, although effects were modest and did not survive correction for multiple comparisons. A key finding is a difference in central-channel contribution patterns between conditions, consistent with memory-related neural modulation. These findings also highlight the potential of covariance-based decoding approaches for probing distributed sleep EEG dynamics, warranting further validation in larger samples.

## 1 Introduction

Newly acquired information must undergo active consolidation during sleep to effectively transition into long-term memory (Brodt et al., 2023; Diekelmann and Born, 2010; Dudai, 2012; Klinzing et al., 2019). This consolidation involves reactivating and strengthening recently formed memory traces, predominantly occurring during non-rapid eye movement (NREM) sleep (Denis and Cairney, 2023). A technique known as targeted memory reactivation (TMR), or cued memory reactivation (Klinzing et al., 2019; Oudiette and Paller, 2013) can selectively enhance this process. In TMR, discrete sensory cues such as odors (Diekelmann et al., 2011; Rasch et al., 2007) or sounds (Antony et al., 2012; Rudoy et al., 2009; Schönauer et al., 2014) are associated with specific learning episodes during wakefulness. Subsequent re-presentation of these sensory cues during sleep selectively reactivates the associated memories, thereby guiding the consolidation process and enhancing memory recall performance upon awakening, even in the absence of the original cues during retrieval (Carbone and Diekelmann, 2024; Klinzing and Diekelmann, 2019).

Auditory cues are frequently preferred in neuroscientific studies of memory reactivation because of their precision and practicality. They allow multiple distinct cues to be presented within a single night, are independent of respiratory cycles, and generate precisely timed neural responses with distinct onsets and offsets (Klinzing and Diekelmann, 2019). In contrast, odor cues offer unique advantages, as they bypass thalamic relays to reach the neocortex and hippocampus more directly and generally do not disrupt sleep (Carskadon and Herz, 2004; Zelano and Sobel, 2005). Additionally, odors do not typically elicit stereotypical evoked responses, thereby reducing potential confounding factors in analyses sensitive to amplitude or signal-to-noise variations. While slow oscillations and spindles, critical components linked to memory consolidation, are readily modulated by auditory cues (Brodt et al., 2023; Klinzing et al., 2019), fewer studies have demonstrated comparable effects using odor-based cues (Bar et al., 2020; Cox et al., 2014; Hauner et al., 2013; Rihm et al., 2016). The variability observed in these effects likely reflects complex interactions among task type, cue modality, and analysis approaches, as well as inconsistencies in spatial, spectral, and temporal features of neural responses (Cox et al., 2014; Klinzing and Diekelmann, 2019; Rihm et al., 2016).

Changes in neural activity resulting from auditory or odor cueing of declarative memories can be effectively measured using electroencephalographic (EEG) recordings. Analyzing EEG data recorded during sleep allows researchers to observe specific brainwave patterns and neural signatures associated with TMR (Creery et al., 2022; Sánchez-Corzo et al., 2024). For instance, with EEG analysis, Sánchez-Corzo et al. (2024) demonstrated that odor cueing during sleep increased centro-parietal sleep spindle rates and slow oscillation amplitudes, and prolonged spindle durations, particularly in frontal regions. Beyond traditional manual selection of EEG features related to sleep to identify neural signatures of TMR, machine learning and deep learning approaches provide efficient and automated alternatives. These computational methods can rapidly uncover additional neural signatures and distinctive patterns associated with TMR, potentially expanding our understanding of the underlying neural processes.

Decoding EEG signals has emerged as an important research domain, with significant advancements achieved through deep learning models tailored specifically to EEG data characteristics (Bozhkov and Georgieva, 2018; Elwasify et al., 2026). However, while deep learning models excel with mid- to large-scale datasets, their effectiveness can diminish when limited data is available. In such cases involving smaller EEG datasets, alternative methods leveraging Riemannian geometry, particularly those implemented in the PyRiemann framework, have demonstrated notable advantages. PyRiemann utilizes covariance matrices of EEG signals, exploiting their intrinsic geometric structure to efficiently extract meaningful features and achieve robust classification performance (Tibermacine et al., 2024). This approach has been rigorously validated across diverse EEG datasets, consistently delivering superior results, particularly in scenarios constrained by limited data availability (Barachant et al., 2013; Congedo et al., 2013). Thus, PyRiemann remains an indispensable tool for EEG researchers working with small datasets where traditional deep learning methods may struggle.

Building on the findings of Sánchez-Corzo et al. (2024), we trained advanced machine-learning classifiers using PyRiemann to distinguish EEG epochs elicited by a declarative memory-associated odor (Odor D) from those elicited by a vehicle control stimulus (sham). Meanwhile, we also conducted parallel classifications between EEG epochs elicited by an odor linked to a motor task (Odor M) and those elicited by vehicle stimulus. While Sánchez-Corzo et al. demonstrated that odor cues associated with declarative memories can modulate sleep oscillatory dynamics, our approach extends these findings by testing whether such modulation is specific to memory reactivation rather than reflecting a general response to task-related odors. To dissociate memory-specific reactivation effects from general sensory or task-related responses, Odor M was included as a cue to be associated with a motor task that did not involve learning (i.e., no sequence learning). This design allowed us to test whether odor-induced modulation of sleep EEG reflects memory reactivation processes rather than a general response to task-associated odors. By contrasting EEG responses collected from Night D (Odor D vs. vehicle) and Night M (Odor M vs. vehicle), we aim to identify neural signatures that uniquely index memory reactivation and consolidation processes. We hypothesized that Odor D would evoke distinctive sleep EEG patterns consistent with memory reactivation, whereas Odor M and the vehicle stimulus would produce less discriminative or less consistent patterns.

To capture the neural dynamics underlying these processes, we analyzed EEG data across multiple frequency bands (fast- and slow-spindle frequencies), spatial channels (all, frontal, central, and posterior channels), and temporal windows (0–2, 0–4, and 0–7 s). This multiscale approach allowed us to examine whether memory reactivation during sleep is expressed within specific spectral, spatial, and temporal domains, thereby offering new insight into the neural mechanisms of odor-cued memory processing during sleep.

## 2 Materials and methods

### 2.1 EEG data description

Sleep EEG data used in this study were collected from 23 healthy young adults (12 females) aged 19–25 years (mean: 22 years, std: 2 years) across two experimental nights using a high-density EEG recording system. The second experimental night was conducted 14–28 days after the first. Further experimental design details can be found in Sánchez-Corzo et al. (2024).

**FIGURE 1:**
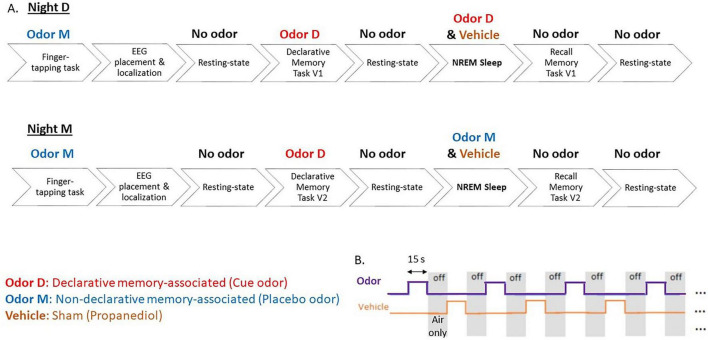
Experimental paradigm used for EEG data collection. **(A)** Participants completed two experimental nights. Each night began with a finger-tapping task accompanied by Odor M, followed by EEG cap placement and localization. Participants then learned a declarative memory task in the presence of Odor D. Afterward, participants slept while EEG was recorded. During deep sleep, olfactory stimulation was administered, alternating between Odor D (Night D) or Odor M (Night M) and an odorless vehicle sham, according to the experimental condition (counterbalanced across participants). Participants were awakened and tested on their recall of the memory task without odor stimulation. **(B)** During NREM sleep, Odor D or M was presented in 15-s intervals alternating with an odorless vehicle, each separated by 15-s breaks (sequence: 15 s odor, 15 s break, 15 s vehicle, 15 s break, etc.).

Figure 1A shows the experimental paradigm of the EEG data used in this study. During each experimental night, participants performed a finger-tapping task with no learnable sequences. During this task, they were stimulated via a face mask with an odor defined as the “Motor task-associated odor” (Odor M). After that, participants performed a declarative memory task, learning the locations of card pairs while being presented with an odor designated as the “Declarative memory task-associated odor” (Odor D). After initial learning, participants completed immediate recall tests to reinforce encoding of card locations. These recall tests were repeated up to five times or until participants correctly recalled over 60% of the card locations.

During subsequent sleep, sleep was continuously monitored online to identify periods of slow-wave sleep (SWS; corresponding to NREM3) for odor stimulation. The experimenter manually initiated cueing once participants entered stable SWS, based on standard polysomnographic features including high-amplitude slow-wave activity and the absence of wake or REM-related features. Stimulation was stopped immediately when participants transitioned out of SWS or awoke. For formal sleep-stage classification, recordings were scored offline in 30-s epochs according to standard criteria, using channels equivalent to C3/C4 referenced to the averaged mastoids together with EOG and EMG-like derivations extracted from the high-density EEG montage. Offline scoring confirmed that cue presentation occurred predominantly during SWS.

As participants entered SWS, they were exposed either to Odor D (Night D) or M (Night M), following a balanced crossover design. Citral and Isobutyraldehyde (Merck Sigma Aldrich) served as Odors D and M, respectively, counterbalanced across participants. Odors were presented during 15-s windows, alternating with an odorless vehicle presentation, each separated by 15-s intervals (Figure 1B). Only air was presented during the 15-s intervals. Stimulation continued until participants exited SWS or woke up, with a maximum sleep session of 90 min, resulting in 40–80 stimulations (58.8+/-11.8) per participant per night.

EEG data were acquired throughout sleep using a 129-channel high-density EEG system (Electrical Geodesics Inc., Eugene, United States) with electrodes placed according to a modified 10–10 system (HydroCel Geodesic Sensor Net). Signals were recorded at a 1,000 Hz sampling rate and referenced online to the vertex electrode. Electrode positions relative to participants’ heads were precisely recorded using optical tracking (Localite TMS Navigator, Localite GmbH, Bonn, Germany). Further tracking details can be found in Sánchez-Corzo et al. (2024).

### 2.2 Preprocessing of EEG data

EEG data during NREM sleep were the focus of analysis in this study. EEG data were preprocessed using the FieldTrip toolbox (Oostenveld et al., 2011). The datasets were first segmented into epochs ranging from -5 to +15 s relative to stimulus onset. Epochs shorter than 15 s in duration (e.g., stimulation errors) were discarded (mean ± SD: 2.46 ± 2.54%). Subsequently, the data were low-pass filtered at 30 Hz using a finite impulse response filter. An additional median filter (order: 30) was applied to suppress occasional non-sinusoidal technical artifacts.

Electrodes located on the face or neck were excluded from further analyses. Noisy EEG channels were visually identified and interpolated using a distance-based weighted average of neighboring channels (mean ± SD interpolated channels: 1.14 ± 2.72%). All epochs were visually inspected for artifacts. Epochs containing widespread artifacts across multiple channels were rejected in their entirety (mean ± SD rejected epochs: 1.67 ± 5.32%). These artifacts typically reflected transient, high-amplitude disturbances affecting a broad spatial extent of the EEG montage, consistent with significant movement or recording-related noise. In contrast, epochs with brief, channel-specific artifacts were retained and corrected using trial-specific channel interpolation (mean ± SD interpolated epochs: 3.28 ± 7.52%).

For subsequent analyses, EEG data were re-referenced to the average signal from both mastoid electrodes. If a mastoid channel (E57 or E100 in the 128-channel Electrical Geodesics Inc. sensor layout) was identified as noisy, it was replaced by the nearest unaffected neighboring electrode based on the native geometry of the electrode net. This occurred in 7 recordings (∼15% of the dataset), with replacement electrodes including E50, E56, E63, E99, E101, and E107. In rare instances where both mastoid channels were affected, both reference channels were replaced accordingly (4.3% of the dataset).

### 2.3 EEG input configurations for classification

**FIGURE 2:**
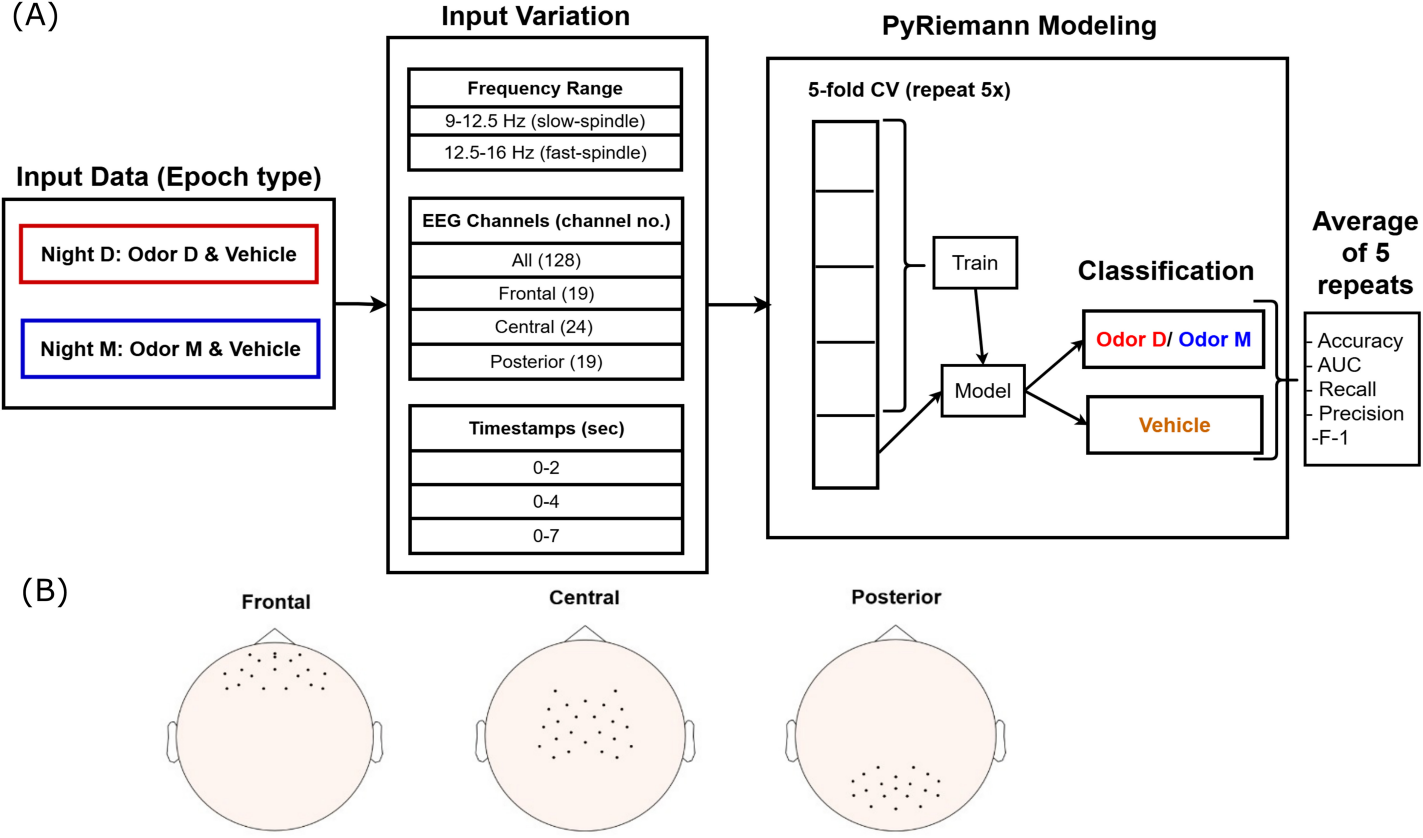
**(A)** Schematic illustration of the EEG epoch classification pipeline employed in this study, highlighting input data, input variation, modeling approach, final classification outcomes, and evaluation metrics. **(B)** Topographic maps of frontal, central, and posterior channel subsets.

To systematically examine odor-related neural signatures and compare classification performance between odor and vehicle (sham) stimulation across nights, EEG epochs from Night D and Night M were analyzed using multiple input configurations (Figure 2). EEG data were filtered into two spindle frequency ranges, fast- (12.5–16 Hz) and slow-spindle (9–12.5 Hz) frequencies, and extracted from predefined spatial electrode subsets (all, frontal, central, and posterior channels). Epochs were segmented into three post-stimulus time windows (0–2, 0–4, and 0–7 s). This factorial design allowed systematic evaluation of temporal, spectral, and spatial contributions to odor-related classification. EEG data were resampled to 256 Hz and preprocessed identically across all conditions prior to classification.

Electrode groupings were defined based on the standard spatial layout of the 128-channel Geodesic Sensor Net and prior region-based parcellation schemes (e.g., Figure 1A in Wen et al., 2016). Electrodes were assigned to regions according to their approximate anatomical scalp locations. This grouping facilitates interpretable comparisons across regions and reduces dimensionality for systematic evaluation of spatial contributions. Fast- and slow-spindle frequency ranges were defined a priori based on commonly reported values in the literature (Andrillon et al., 2011; Mölle et al., 2011) and were applied uniformly across participants. Three post-stimulus time windows were defined to capture the temporal evolution of neural responses to odor cueing, from early to sustained effects. This choice was informed by a prior work showing that odor-induced modulation of sleep oscillations, including spindle activity and slow oscillations, can extend for several seconds following stimulus onset (Sánchez-Corzo et al., 2024).

The analysis configurations are not fully independent: time windows are nested, and channel subsets partially overlap. While the frequency bands are non-overlapping (9–12.5 vs. 12.5–16 Hz), they are not entirely independent, as spindle activity may span adjacent frequency ranges and filtering may introduce some spectral leakage. The nested time windows allow assessment of whether discriminative patterns are transient or sustained, while overlapping channel subsets enable comparison between global and region-specific contributions. In contrast, the non-overlapping frequency bands allow differentiation between fast and slow spindle activity. Accordingly, comparisons across configurations are interpreted descriptively to assess the robustness and consistency of effects, rather than as independent statistical tests.

### 2.4 Covariance-based Riemannian classification

**FIGURE 3:**
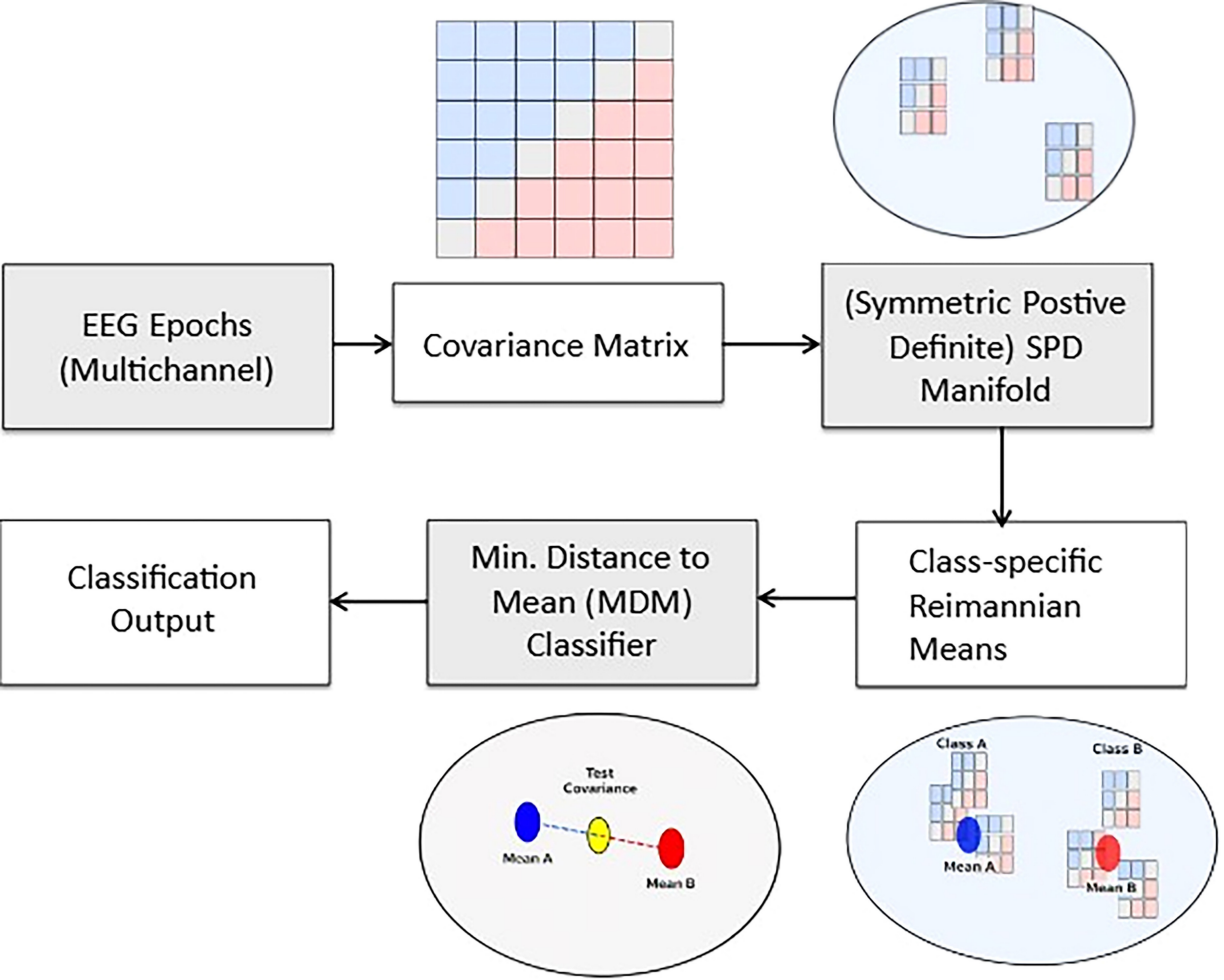
Schematic diagram illustrating the workflow of PyRiemann classification. EEG epochs are first converted into covariance matrices, which are then represented as points on a symmetric positive definite manifold. Class-specific Riemannian means are computed, followed by classification using the minimum-distance-to-mean approach to determine epoch class labels (e.g., odor vs. vehicle).

Classification was performed using PyRiemann (Barachant et al., 2026), a Python package designed for multivariate analysis of biosignals using Riemannian geometry. For each EEG epoch, spatial covariance matrices were computed across channels and treated as symmetric positive-definite (SPD) matrices (Figure 3). Classification was performed using the Minimum Distance to Mean (MDM) classifier with the affine-invariant Riemannian metric (Barachant et al., 2013). This approach is well-suited for EEG decoding with limited trial counts and avoids overparameterization common in high-dimensional models. All analyses were conducted using a within-participant classification framework. For Night D, EEG epochs elicited by Odor D were classified against vehicle, and for Night M, EEG epochs elicited by Odor M were classified against vehicle. Classification was repeated independently for each combination of frequency band, time window, and channel subset.

Classification was performed within participant and within night, ensuring that each comparison was made under the same recording conditions. This design reduces sensitivity to nonspecific between-night variations such as electrode impedance or global signal scaling. In addition, EEG segments were band-pass filtered, and covariance matrices were computed directly from these signals, capturing relative inter-channel relationships rather than absolute signal amplitudes. The use of covariance-based features emphasizes relative relationships between channels and reduces sensitivity to global amplitude differences.

### 2.5 Cross-validation procedure

Models were trained and evaluated separately for each participant. For each participant and condition, EEG epochs were partitioned into five stratified folds, each balanced for odor and vehicle trials. In each cross-validation iteration, four-folds (80%) were used to train the classifier, and the remaining fold (20%) was used for testing. This process was repeated five times such that each fold served once as the test set (five-fold cross-validation). To obtain stable estimates of classification performance, the entire five-fold cross-validation procedure was repeated five times using different random fold assignments. Classification accuracy was computed for each fold and repetition and then averaged to yield a mean accuracy per participant for each analysis configuration. This procedure was performed independently for each combination of channel subset (4 levels), time window (3 levels), and night (2 levels), separately for signals filtered in the fast and slow frequency ranges. In total, each participant underwent 600 evaluations (4 channel subsets × 3 time windows × 2 nights × 25). By systematically varying training and testing partitions through repeated cross-validation, this procedure provided conservative estimates of classification accuracy, thereby reducing variance and mitigating potential overfitting. Although computationally intensive, this approach enhanced the statistical rigor and reliability of the reported performance metrics.

### 2.6 Permutation testing

Chance-level performance was estimated using a permutation procedure applied independently for each participant and condition. Class labels were randomly permuted across trials, thereby destroying the relationship between the data and labels while preserving class balance. For each participant, 1,000 permutations were performed. This number of permutations provides a stable estimate of the null distribution while balancing computational cost and precision. For each permutation, the full cross-validation procedure (5-fold, 5 repeats) was repeated, and performance was averaged across folds and repeats to yield a single permutation score. This resulted in a participant-specific null distribution of decoding performance. To ensure a fair comparison between observed and null performance, identical cross-validation splits were used for both real and permuted analyses. Cross-validation indices were generated once per participant and reused across all permutations.

Participant-level permutation *p*-values were computed as:


$$p=\frac{1+♯\left(\mathrm{perm}\geq \mathrm{real}\right)}{N_{\text{perm}}+1}$$

where *N*_perm_ = 1000.

### 2.7 Group-level statistical analysis

Group-level inference was performed using paired statistical tests across participants. For each condition (night × channel subset × time window), decoding performance was compared against permutation-based chance using participant-level summary values, in which the observed decoding performance was contrasted with the mean of the corresponding permutation-derived null distribution. The null hypothesis stated that the mean difference between real and permutation performance equals zero (H0: mean (real - permutation) = 0). This analysis yielded a t-statistic and corresponding *p*-value for each condition. Multiple comparisons across all tested conditions (4 channel subset × 3 time windows × 2 nights = 24 tests) were controlled using the Benjamini–Hochberg false discovery rate (FDR) procedure at α = 0.05. In this framework, permutation testing is used to estimate a participant-specific chance performance, while group-level statistical inference is performed across participants using these summary values. The paired *t*-test was used to evaluate whether observed decoding performance consistently exceeded permutation-derived chance across participants, treating participants as independent observations.

The primary hypothesis that decoding performance differs between nights was evaluated using participant-level normalized scores computed as the difference between real and permutation performance (normalized = real - permutation). For each channel subset and time window, normalized decoding performance was compared between D and M nights using paired *t*-tests across participants. Resulting *p*-values were corrected for multiple comparisons across all channel subset × time combinations using FDR. Additional descriptive measures were computed to characterize the distribution of effects across participants, including the mean and standard error of decoding performance, the number of participants with individually significant permutation results (*p* < 0.05), and the proportion of participants showing higher normalized performance in Night D relative to Night M. Uncorrected *p*-values are reported for completeness and are used in figures to indicate exploratory trends.

### 2.8 Channel-level contribution analysis

**FIGURE 4:**
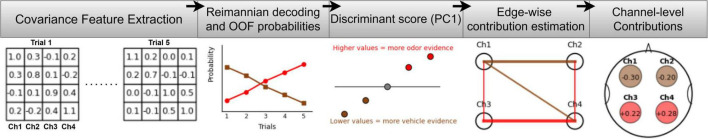
Schematic overview of the channel-level contribution analysis. Trial-wise covariance matrices were computed from EEG segments and classified using a Riemannian decoder to obtain out-of-fold probability estimates. These probabilities were projected onto a one-dimensional discriminant axis, and edge-wise covariance features were correlated with the resulting trial-level scores. Edge contributions were then redistributed to their incident channels, yielding signed channel-level contribution maps in which positive values indicate odor-supporting covariance patterns and negative values indicate vehicle-supporting covariance patterns.

We performed a post hoc interpretability analysis to identify which spatial covariance patterns contributed to classification, linking classifier outputs to covariance features and channel-level contributions. The complete process is illustrated in Figure 4.

#### 2.8.1 Covariance feature extraction

For each participant, trial-wise multichannel EEG segments were extracted within the defined time windows and frequency bands. For each trial, spatial covariance matrices were computed using the Oracle Approximating Shrinkage (OAS) estimator (Chen et al., 2010), providing a second-order statistical characterization of linear co-fluctuation structure across EEG channels. These covariance matrices were regularized to ensure strict positive definiteness, yielding SPD representations suitable for Riemannian decoding.

#### 2.8.2 Riemannian decoding and discriminant score derivation

Covariance matrices were classified using the Riemannian MDM classifier as described above. To obtain unbiased trial-level outputs, stratified cross-validation was used to obtain out-of-fold (OOF) posterior probabilities for each trial yielding probability vectors [p(vehicle), p(odor)] that reflect classifier confidence. To derive a one-dimensional measure of classifier output, these probability vectors were projected onto a single discriminant axis using principal component analysis (PCA). The first principal component (PC1) captured the dominant variation in classifier predictions, and its sign was aligned such that higher values consistently reflected stronger evidence for the odor condition.

#### 2.8.3 Reliability weighting and permutation testing

To account for variability in decoding performance across participants, we estimated participant-level reliability using permutation testing. For each participant, class labels were shuffled to generate a null distribution of the receiver operating characteristic curve (AUC) values, and a permutation-based *p*-value (*p*_*perm*_) was computed. For interpretability analyses, participants were weighted using w = 1 - *p*_*perm*_, such that more reliable decoders contributed more strongly to group-level estimates.

#### 2.8.4 Channel-level contribution estimation

We used a Haufe-style activation pattern approach (Haufe et al., 2014) to link classifier output back to covariance features. Covariance matrices were vectorized by extracting upper-triangular elements, yielding edge-wise features representing pairwise covariance between channels. These features were z-scored across trials and correlated with the corresponding PC1 discriminant scores, producing edge-wise contribution values that quantify the association between covariance features and classifier output. Edge-wise contributions were then mapped to channels by assigning half of each edge’s contribution to its two constituent channels. The resulting channel-level contributions were L1-normalized, weighted by participant reliability, and averaged across participants to obtain group-level maps.

#### 2.8.5 Interpretation of signed channel contributions

Because the discriminant axis was oriented such that higher values corresponded to the odor condition, positive channel contributions indicate covariance patterns that support odor-related classification, whereas negative contributions indicate patterns supporting vehicle classification. These contributions reflect associations with classifier output rather than local signal amplitude. This interpretation follows established guidelines for decoding models (Grootswagers et al., 2017; Haufe et al., 2014; King and Dehaene, 2014). Aggregation of edge-wise contributions to channels provides a summary measure of each channel’s involvement in the discriminative covariance structure, analogous to node-strength measures in network neuroscience (Rubinov and Sporns, 2010).

#### 2.8.6 Topographic visualization

Channel-level contribution values were visualized as scalp topographic maps separately for frequency band (fast- vs. slow-spindle frequencies) and time window (0–2, 0–4, and 0–7 s). Red and blue colors indicate channels whose covariance patterns preferentially support odor-related classification, whereas brown colors indicate channels whose covariance patterns preferentially support vehicle classification. These maps provide a spatially interpretable representation of the network-level features driving classification performance.

## 3 Results

### 3.1 Decoding performance relative to permutation-based chance

**FIGURE 5:**
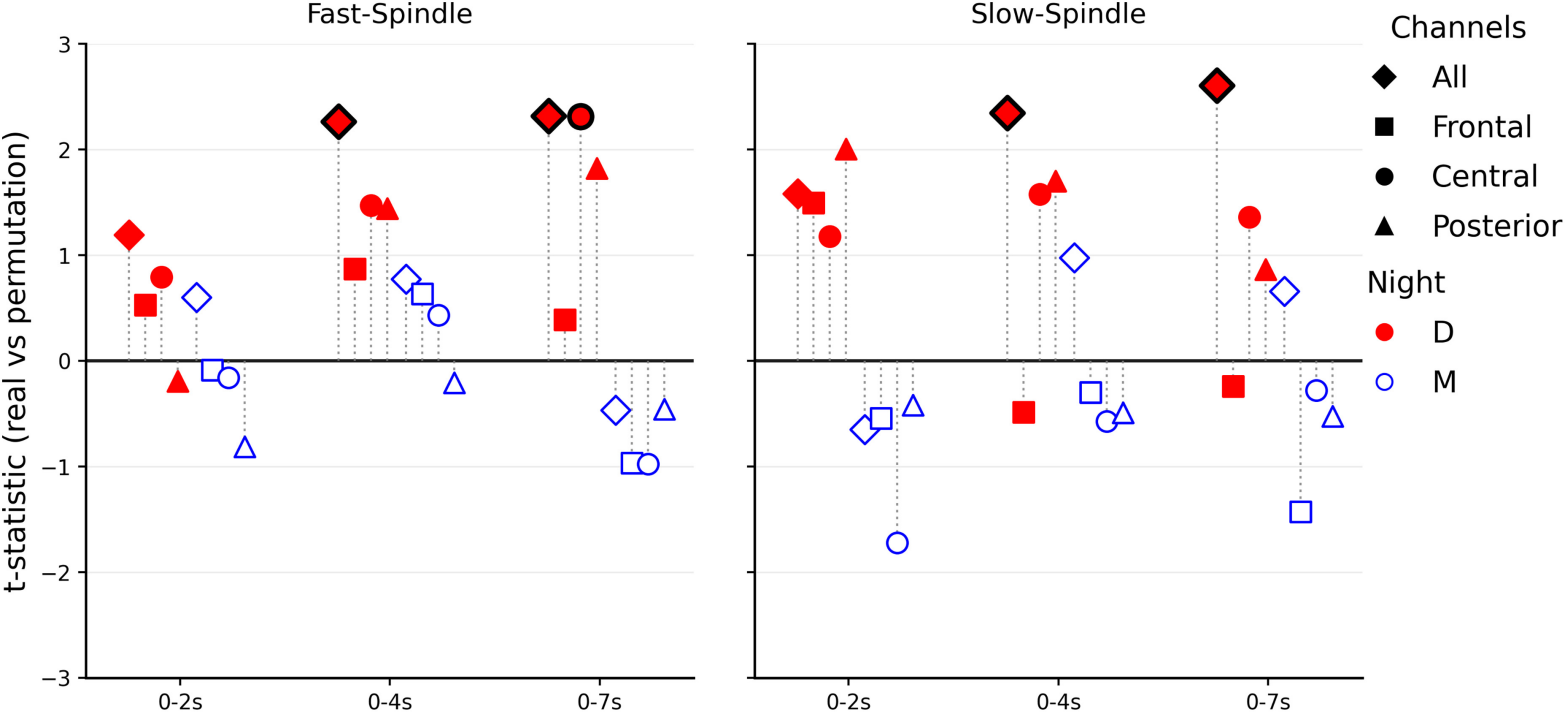
Decoding performance relative to permutation-derived chance across fast (left) and slow (right) spindle frequency bands. Each lollipop represents the group-level *t*-statistic for the comparison between real decoding accuracy and permutation-based chance for a given channel subset, time window, and night. Marker shape indicates channel subset, and marker fill indicates night (D: filled red; M: open blue). Black outlines denote uncorrected *p* < 0.05. Positive values indicate above-chance decoding.

Decoding performance was evaluated relative to permutation-derived chance to establish the presence of above-chance classification prior to comparing normalized differences between conditions. Across 23 participants, the mean total number of trials per participant was 57.65 ± 12.87 for Night D vs. vehicle and 59.91 ± 10.76 for Night M vs. vehicle, with equal representation of odor and vehicle trials in both analyses. Figure 5 shows that decoding performance varied as a function of spindle frequency, night, region, and time window. Across both fast and slow spindle analyses, the strongest effects were observed in Night D, whereas Night M showed weak or absent evidence for above-chance decoding.

For the fast spindle analysis, three conditions reached uncorrected significance (*p* < 0.05), all in Night D: the all-channel set at 0–4 s (*t* = 2.265, *p* = 0.034, normalized accuracy = 0.040), the all-channel set at 0–7 s (*t* = 2.317, *p* = 0.030, normalized accuracy = 0.034), and the central region at 0–7 s (*t* = 2.314, *p* = 0.030, normalized accuracy = 0.032). An additional trend-level effect was observed in the posterior region at 0–7 s (*t* = 1.823, *p* = 0.082, normalized accuracy = 0.022). Across all conditions for fast-spindle band and in Night D, the mean *t*-statistic was 1.269, whereas the corresponding value for Night M was -0.139, indicating that the fast-spindle effects were specific to Night D.

For the slow spindle analysis, two conditions reached uncorrected significance, again exclusively in Night D: the all-channel set at 0–4 s (*t* = 2.348, *p* = 0.028, normalized accuracy = 0.037) and the all-channel set at 0–7 s (*t* = 2.605, *p* = 0.016, normalized accuracy = 0.038). A trend-level effect was observed in the posterior region at 0–2 s (*t* = 2.004, *p* = 0.058, normalized accuracy = 0.039). In contrast, Night M showed no uncorrected significant effects; the only trend-level effect was a negative deviation in the central region at 0–2 s (*t* = -1.722, *p* = 0.099, normalized accuracy = -0.022). Across all conditions for slow-spindle band and Night D, the mean *t*-statistic was 1.334, compared with -0.440 for Night M.

Direct comparison of the fast and slow spindle results indicated partially distinct spatial-temporal profiles. Fast-spindle effects were relatively stronger in the central region at longer durations, particularly for Night D at 0–7 s (fast: *t* = 2.314, slow: *t* = 1.362), whereas slow-spindle effects were relatively stronger in posterior regions at shorter durations, most notably for Night D in the posterior region at 0–2 s (fast: *t* = -0.190, slow: *t* = 2.004). Despite these condition-specific trends, no effect survived false discovery rate correction. Overall, these findings indicate a consistent tendency for decoding performance to exceed permutation-derived chance in Night D, with effects concentrated in the all-channel set and central region at longer durations for fast spindles, and in the all-channel set and posterior region for slow spindles. By contrast, Night M showed little evidence for above-chance decoding in either frequency band.

### 3.2 Normalized decoding differences between D and M conditions

**FIGURE 6:**
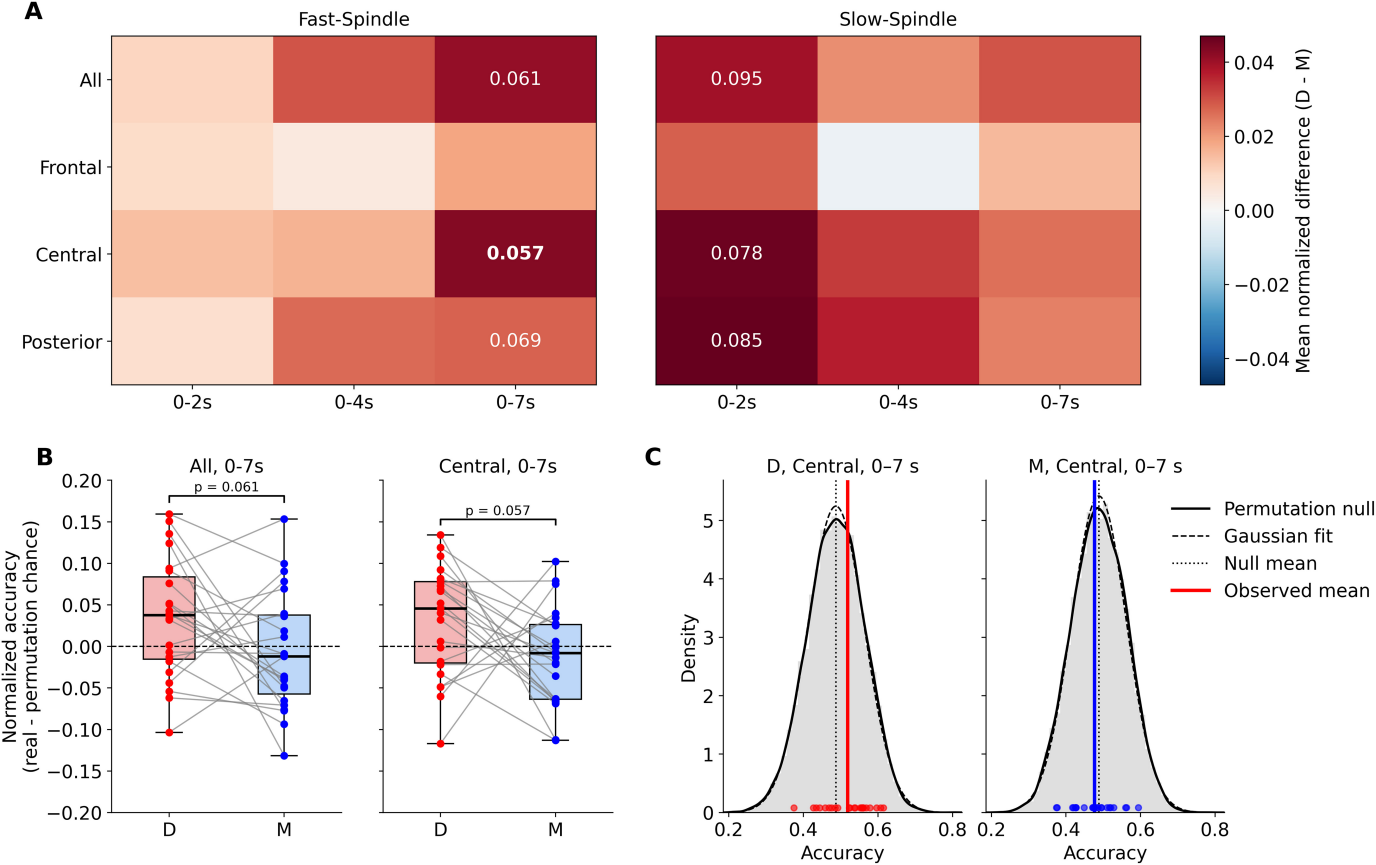
Mean participant-level normalized decoding differences (D—M) across channel subsets and time windows for fast (left) and slow (right) spindle analyses. **(A)** Heatmaps show the average normalized difference for each condition; cell values indicate uncorrected *p*-values (*p* < 0.10 shown). **(B)** Paired participant-level normalized decoding performance for all and central channel subset at 0–7 s (fast spindle), with lines connecting individual participants and brackets indicating uncorrected *p*-values. **(C)** Observed decoding accuracy relative to the permutation-derived null distribution for the central channel subset at 0–7 s (fast spindle). Gray histograms and black curves show the pooled null distribution; colored vertical lines indicate observed group means.

Figure 6A shows that normalized decoding performance (real accuracy minus permutation-derived chance) was generally higher in Night D than in M, although the magnitude and consistency of this effect varied across regions and time windows. In the fast spindle analysis, the largest differences were observed at longer durations. At 0–7 s, decoding was higher in Night D than M for the all-channel set (Δ = 0.041 ± 0.100, *p* = 0.061) and central region (Δ = 0.043 ± 0.103, *p* = 0.057), with a similar trend in posterior regions (Δ = 0.028 ± 0.069, *p* = 0.069). Effects at shorter durations (0–2 s, 0–4 s) were smaller and less consistent across regions. In contrast, the slow spindle analysis showed weaker and less structured differences. The largest effects were observed at 0–2 s in central (Δ = 0.046 ± 0.120, *p* = 0.078) and posterior regions (Δ = 0.047 ± 0.125, *p* = 0.085), with no clear pattern at longer durations. No condition survived false discovery rate correction across region × time comparisons.

Figure 6B next examined whether the observed group-level differences reflected consistent effects across participants. For the fast spindle analysis at 0–7 s, normalized decoding was higher in Night D than M for the all-channel set (D: 0.034 ± 0.071; M: -0.007 ± 0.071; Δ = 0.041 ± 0.100, *p* = 0.061), with 16/23 participants (69.6%) showing a D > M effect. A similar pattern was observed in the central region (D: 0.032 ± 0.066; M: -0.012 ± 0.057; Δ = 0.043 ± 0.103, *p* = 0.057), where 15/23 participants (65.2%) exhibited higher decoding in Night D than in M. These results indicate that the effect of Odor D was distributed across participants rather than driven by a small number of outliers, although the magnitude of the effect remains modest.

Figure 6C further characterizes the observed effects by comparing decoding performance to permutation-derived null distributions for the central region at 0–7 s. In Night D, mean observed accuracy (0.519 ± 0.062) exceeded the mean of the null distribution (0.487 ± 0.076), indicating a shift toward above-chance performance. In contrast, Night M showed no such shift (observed: 0.477 ± 0.056; null: 0.488 ± 0.074). For visualization purposes, permutation-derived decoding scores were pooled across participants to illustrate the null distribution of classification performance; these plots are descriptive and not used for statistical inference.

Together, these findings suggest a modest and spatially localized tendency for decoding performance to be higher in Night D than in M, particularly in central regions at longer time windows during the fast spindle analysis. However, these effects did not survive correction for multiple comparisons.

### 3.3 Central-channel contribution for Odor D versus vehicle classification

**FIGURE 7:**
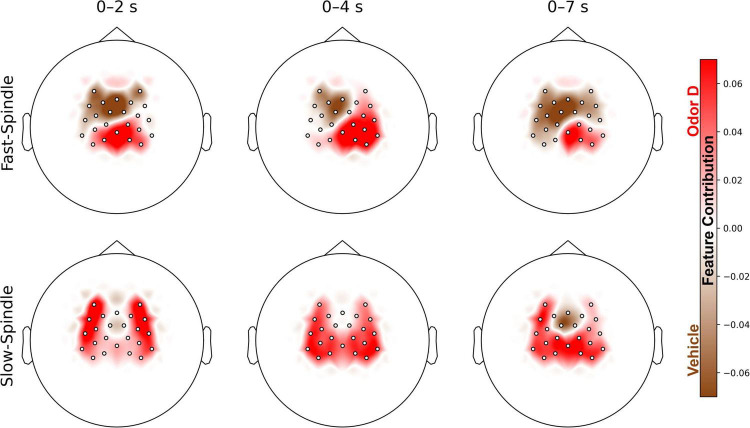
Central-channel contributions to odor-based classification in spindle-band EEG (Odor D vs. vehicle) were predominantly positive across frequency bands and time windows. Topographic maps show channel-level feature contributions derived from covariance-based Riemannian decoding for Odor D vs. vehicle, separately for fast- (12.5–16 Hz) and slow-spindle bands (9–12.5 Hz) in the first and second rows, respectively, and across post-stimulus time windows (0–2, 0–4, and 0–7 s). Positive values (red) indicate covariance patterns that support classification toward the Odor D condition, whereas negative values (brown) indicate patterns that support classification toward the vehicle condition.

Figure 7 shows central channel-level contribution maps for classification between Odor D and vehicle, separately for fast- and slow-spindle frequency bands and across post-stimulus intervals (0–2, 0–4, and 0–7 s). The fast-spindle band across time windows showed a localized covariance pattern, with positive contributions linked to Odor D over posterior-central channels and more negative contributions with the vehicle over anterior-central channels. In the slow-spindle band, early windows showed broadly positive contributions across central channels and a weak negative contribution distributed sparsely.

### 3.4 Central-channel contribution for Odor M versus vehicle classification

**FIGURE 8:**
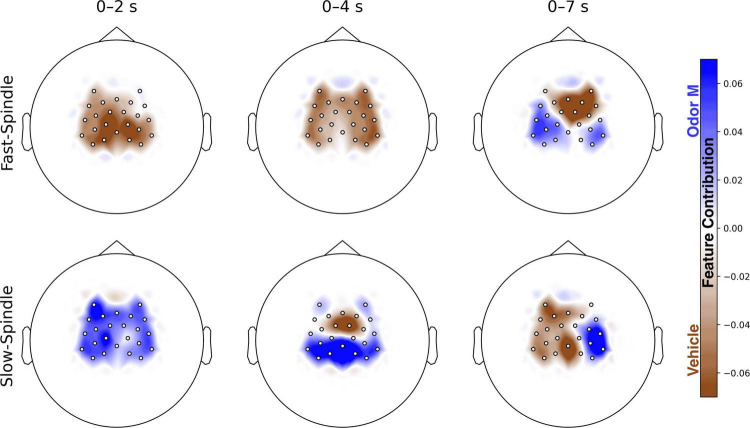
Central-channel contributions to odor-based classification in spindle-band EEG (Odor M vs. vehicle) exhibited negative or mixed contributions across frequency bands and time windows. Topographic maps show channel-level feature contributions derived from covariance-based Riemannian decoding for Odor M vs. vehicle, separately for fast- (12.5–16 Hz) and slow-spindle bands (9–12.5 Hz) in the first and second rows, respectively, and across post-stimulus time windows (0–2, 0–4, and 0–7 s). Positive values (blue) indicate covariance patterns that support classification toward the Odor M condition, whereas negative values (brown) indicate patterns that support classification toward the vehicle condition.

Figure 8 shows the corresponding contribution maps for classification between Odor M and vehicle. In contrast to Figure 7, the fast-spindle band exhibited predominantly negative contributions associated with the vehicle across central channels in early time windows, which later became more pronounced over anterior-central regions. In the slow-spindle band, positive contributions linked to Odor M were observed in the 0–2 s window, followed by a more spatially localized pattern with both positive and negative contributions at later intervals.

## 4 Discussion

Our study used within-participant, covariance-based EEG decoding to examine neural responses to odor cueing during sleep under two conditions: declarative odor cueing (Odor D vs. vehicle during Night D) and non-declarative odor cueing (Odor M vs. vehicle during Night M). Across spindle frequency bands, spatial channel subsets, and post-stimulus time windows, decoding performance showed night-dependent patterns, with a tendency for higher decoding in Night D condition relative to Night M. These effects were primarily observed in the central and posterior regions, and across all channels, whereas frontal regions showed weaker and less consistent effects. Although these differences did not survive correction for multiple comparisons, they were consistently observed across analyses, suggesting a modest but structured distinction in neural responses to declarative versus non-declarative odor cueing.

### 4.1 Condition-dependent decoding patterns during declarative cueing

Decoding performance showed condition-dependent patterns, with a tendency for higher decoding in Night D compared to M, particularly in central and posterior regions and at longer time windows. This pattern was observed at the group level using normalized decoding performance (real accuracy minus permutation-derived chance) and was supported by consistent participant-level differences between conditions.

In the fast spindle band, the strongest effects were observed at longer time windows (Figure 6A, left), particularly in the central region with trends in posterior regions. In contrast, the slow spindle band showed a different temporal profile, with relatively stronger effects at earlier time windows (Figure 6A, right). These findings suggest that spindle-band activity over central and posterior areas contains relatively greater discriminative information for distinguishing Odor D from vehicle stimulation compared to Odor M, whereas frontal regions showed weaker and less consistent effects. Although these differences were modest in magnitude and did not survive correction for multiple comparisons, they were consistently observed across analyses and across participants. These findings are consistent with prior work demonstrating enhanced fast spindle activity in centro-parietal regions following odor cueing of declarative memories during sleep (Sánchez-Corzo et al., 2024). Fast spindles over central regions have been linked to hippocampo-cortical communication and the consolidation of declarative memories, and may therefore provide a neural substrate for the selective reactivation of odor-associated memory representations (Klinzing et al., 2019; Kumral et al., 2023). Posterior cortices, including parietal and occipital regions, are known to contribute to sensory processing and spatial aspects of memory (Baena et al., 2024; Cabeza et al., 2008) and have been implicated in sleep-dependent memory reactivation through coordinated oscillatory activity involving spindles, slow oscillations, and hippocampal ripples (Klinzing et al., 2019; Rasch and Born, 2013). The enhanced involvement of posterior channels during declarative odor cueing may therefore reflect the selective reactivation of sensory memory representations linked to the odor cues during sleep.

### 4.2 Central-channel covariance patterns during odor cueing

Channel-level contribution topographies were examined to assess modulation associated with odor cueing during sleep. Classifier-weighted channel contributions capture condition-specific discriminative modulation by identifying channels whose covariance features are systematically associated with the discriminative axis separating odor and vehicle conditions. In this way, classifier-weighted contributions isolate modulation of spindle-band covariance structure associated with odor presentation, which may reflect odor-induced memory reactivation.

A key finding of this study is the dissociation in central-channel contribution patterns between declarative and non-declarative odor cueing. Across conditions, differences in contribution patterns between declarative (Odor D) and non-declarative (Odor M) cueing were evident within central channels. In the fast spindle band, Odor D was associated with a more spatially structured pattern, with positive contributions over posterior-central channels and opposing contributions over anterior-central regions, suggesting a more organized covariance structure supporting classification. In contrast, Odor M showed more variable contribution patterns, with contributions more strongly associated with the vehicle condition in early time windows. In the slow spindle band, both conditions showed more diffuse and temporally variable patterns, although Odor D exhibited sustained positive contributions across time windows compared to Odor M. Together, these findings support the view that declarative memory reactivation during sleep is associated with more structured and temporally stable large-scale EEG dynamics than non-declarative cueing, consistent with the proposed role of spindle-band activity in coordinating hippocampo–cortical communication during memory consolidation (Klinzing et al., 2019; Rasch and Born, 2013; Staresina et al., 2015).

It is important to note that the channel contribution maps were derived separately for the Odor D vs. vehicle and Odor M vs. vehicle decoding problems, and therefore reflect condition-specific representations of discriminative covariance structure. These contributions depend on the underlying covariance patterns, classifier decision boundaries, and normalization within each model, and are thus not defined on a common absolute scale across conditions. Consequently, direct subtraction or statistical comparison between Odor D and Odor M contribution values is not straightforward to interpret, as any observed differences may arise from both neural effects and model-specific scaling.

Accordingly, statistical inference in this study is based on permutation-derived contrasts between each odor condition and vehicle, along with normalized differences (D-M) computed on a common scale. This provides a more interpretable framework for comparison, as shown in Figures 5, 6. Within this context, channel contribution maps serve as descriptive visualizations of the spatial distribution of discriminative covariance patterns within each condition. When the analysis is restricted to participants with higher decoding performance, these spatial patterns become more pronounced (Supplementary Figures S1, S2), illustrating a stronger contrast between the Odor D and Odor M patterns; however, all primary analyses are conducted on the full cohort to avoid selection bias. Overall, this approach supports a careful interpretation of condition-specific covariance patterns while preserving statistical rigor.

### 4.3 Complexity and variability of EEG patterns during memory reactivation

EEG-based analyses of sleep dynamics during memory reactivation are inherently challenging due to the substantial variability and complexity of sleep-related neural events. Rather than relying on highly consistent, event-locked neural signatures, an informative approach is to examine system-level differences by comparing neural dynamics associated with targeted memory reactivation to those elicited by control conditions. This perspective acknowledges that key sleep events, such as spindles, are temporally sparse and variable across trials and individuals, complicating the identification of stable, time-locked neural markers.

In the present study, we addressed these challenges by systematically contrasting EEG dynamics elicited by an odor associated with declarative memory reactivation (Odor D vs. vehicle) with those elicited by a non-declarative odor (Odor M vs. vehicle). This relative comparison allowed us to isolate neural dynamics specific to targeted memory reactivation, rather than to odor presentation per se. By prioritizing condition-dependent differences over absolute neural patterns, our analyses revealed consistent classification effects unique to Odor D and highlighted spatially specific spindle-band contributions. This system-level decoding framework provides a robust approach for probing memory reactivation processes during sleep despite substantial inter- and intra-individual variability.

The present findings also highlight a methodological contribution. Although the observed effects did not survive correction for multiple comparisons, the analysis reveals consistent patterns that can be captured using covariance-based decoding approaches. In particular, the use of Riemannian geometry-based representations of EEG covariance structure provides an interpretable framework for characterizing memory-related neural dynamics beyond simple amplitude-based measures. Given the substantial variability inherent in sleep EEG, such approaches may be especially valuable for detecting structured differences at the system level. With larger sample sizes or more constrained experimental designs, this framework may provide a sensitive and scalable approach for studying memory reactivation during sleep.

### 4.4 Limitations

Several limitations should be considered when interpreting these findings. Memory reactivation during sleep is a dynamic and sparsely occurring process, and the neural signatures of replay may vary in latency (potentially because of the lack of control in the exact time of odor sensing), frequency, and spatial distribution across individuals and trials. As a result, decoding performance is constrained by imperfect temporal alignment and by uncertainty in trial-level labels, which are inferred indirectly rather than observed directly. These factors likely contribute to reduced decoding accuracy and limit generalization across participants. Although our within-participant decoding approach mitigates some sources of variability, developing participant-independent models of memory reactivation from scalp EEG remains challenging.

Although condition-dependent patterns were observed across analyses, none of the effects survived correction for multiple comparisons, and the findings should therefore be interpreted as reflecting a modest but consistent pattern. The observed differences were modest in magnitude, reflecting relatively small deviations in normalized decoding performance between conditions. Such subtle effects are consistent with the challenges of detecting memory-related neural modulation during sleep but may require larger samples or more targeted experimental designs to establish robust statistical significance. The factorial design spanning multiple regions, time windows, and frequency bands further increases the number of comparisons, and although this was addressed using FDR correction, the exploratory nature of the analysis limits the strength of inferential conclusions.

Channel contribution maps should be interpreted as descriptive representations of condition-specific covariance structure rather than direct statistical contrasts between conditions. In addition, the present analysis is based on within-participant decoding, which captures participant-specific covariance structure but does not directly assess generalization across individuals. Future work may benefit from larger and more heterogeneous datasets, improved inter-participant alignment strategies, and multimodal recordings that provide more precise markers of replay-related events. Together, these advances will be critical for improving the robustness and generalizability of decoding approaches targeting sleep-dependent memory processes. Moreover, although classification was performed within participant and within night to control for recording variability, differences in data quality across nights could still, in principle, influence decoding performance. However, the use of within-night contrasts and covariance-based features is expected to mitigate this effect.

At the group level, permutation-derived null distributions were summarized by their mean for each participant, and statistical inference was performed using paired tests across participants. This approach does not explicitly account for the variability of the null distribution and therefore represents a simplification relative to a fully permutation-based framework. This simplification may be particularly relevant in the context of a modest sample size and substantial inter-individual variability, where a fully permutation-based group-level approach would be more difficult to implement and interpret robustly.

In addition, behavioral performance was not reported in this study because the same experimental paradigm (Figure 1A) has been reported previously (Sánchez-Corzo et al., 2024). In the above study, memory retention was assessed as the percentage of correctly recalled object locations after sleep relative to pre-sleep performance. While the odor cueing manipulation elicited robust neural effects during SWS, the behavioral benefits of TMR were not consistently observed across participants. Therefore, the present study focuses on the neural discriminability of cueing conditions and does not directly address brain-behavior relationships.

## Data Availability

The raw data supporting the conclusions of this article will be made available by the authors, without undue reservation.
