# Supplementary material for: Covariance-based analysis of spindle-band EEG during declarative and non-declarative odor cueing in sleep
Source: Front Neurosci. 2026 Jun 18;20:1810323. doi: 10.3389/fnins.2026.1810323 (PMC13323133; doi:10.3389/fnins.2026.1810323)
Supplement: Supplementary file 1 [file Data_Sheet_1.docx]

***Supplementary Material***

1. **Supplementary Figures and Tables**

**1.1 Supplementary Figures**


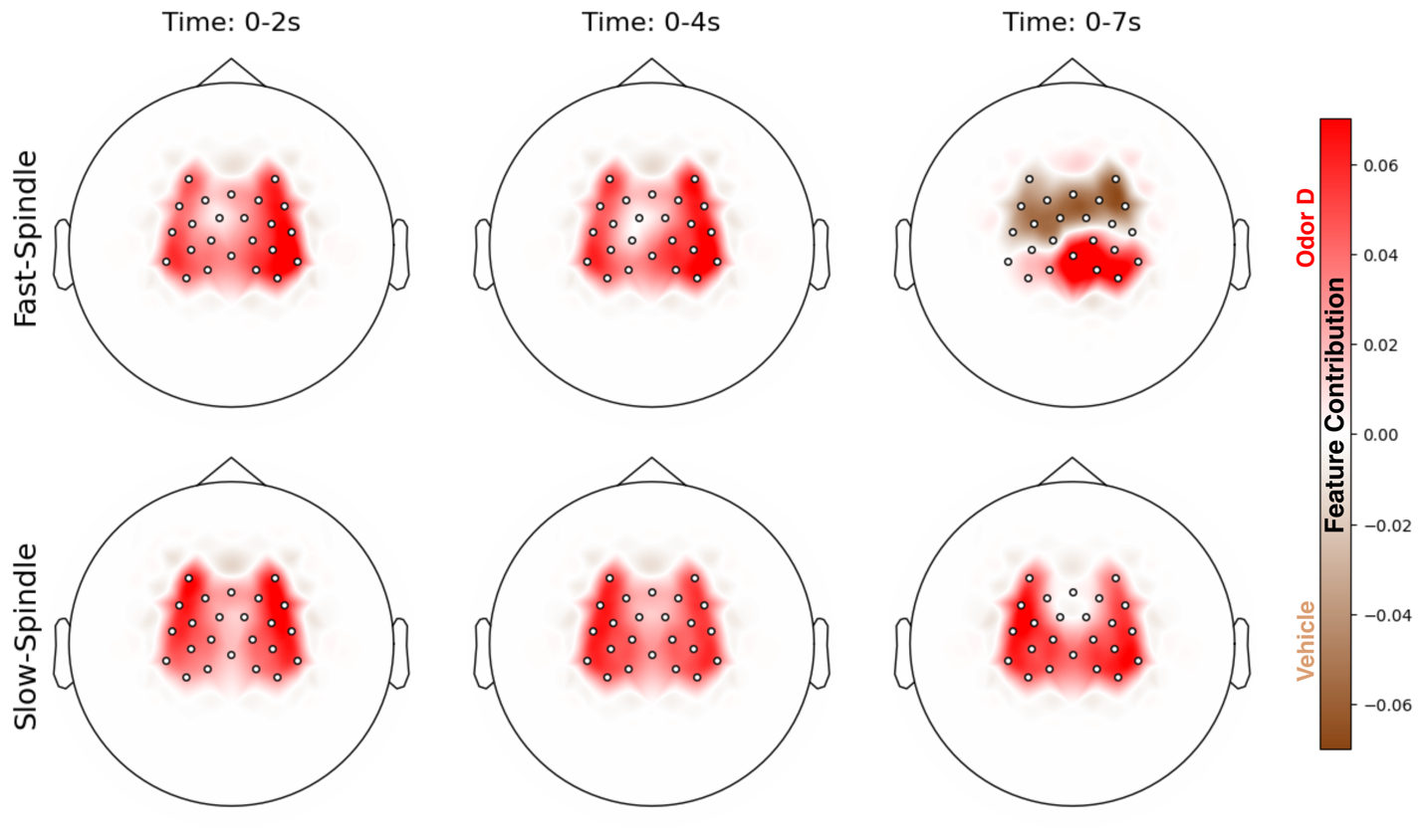


**Supplementary Figure S1**. Selected participants where the mean classification accuracy exceeded the chance level (0.5). Central-channel contributions to odor-based classification in spindle-band EEG (Odor D vs. vehicle) were predominantly positive across frequency bands and time windows. Topographic maps show channel-level feature contributions derived from covariance-based Riemannian decoding for Odor D vs. vehicle, separately for fast- (12.5–16 Hz) and slow-spindle bands (9–12.5 Hz) in the first and second rows, respectively, and across post-stimulus time windows (0–2, 0–4, and 0–7 s). Positive values (red) indicate covariance patterns that support classification toward the Odor D condition, whereas negative values (brown) indicate patterns that support classification toward the vehicle condition.


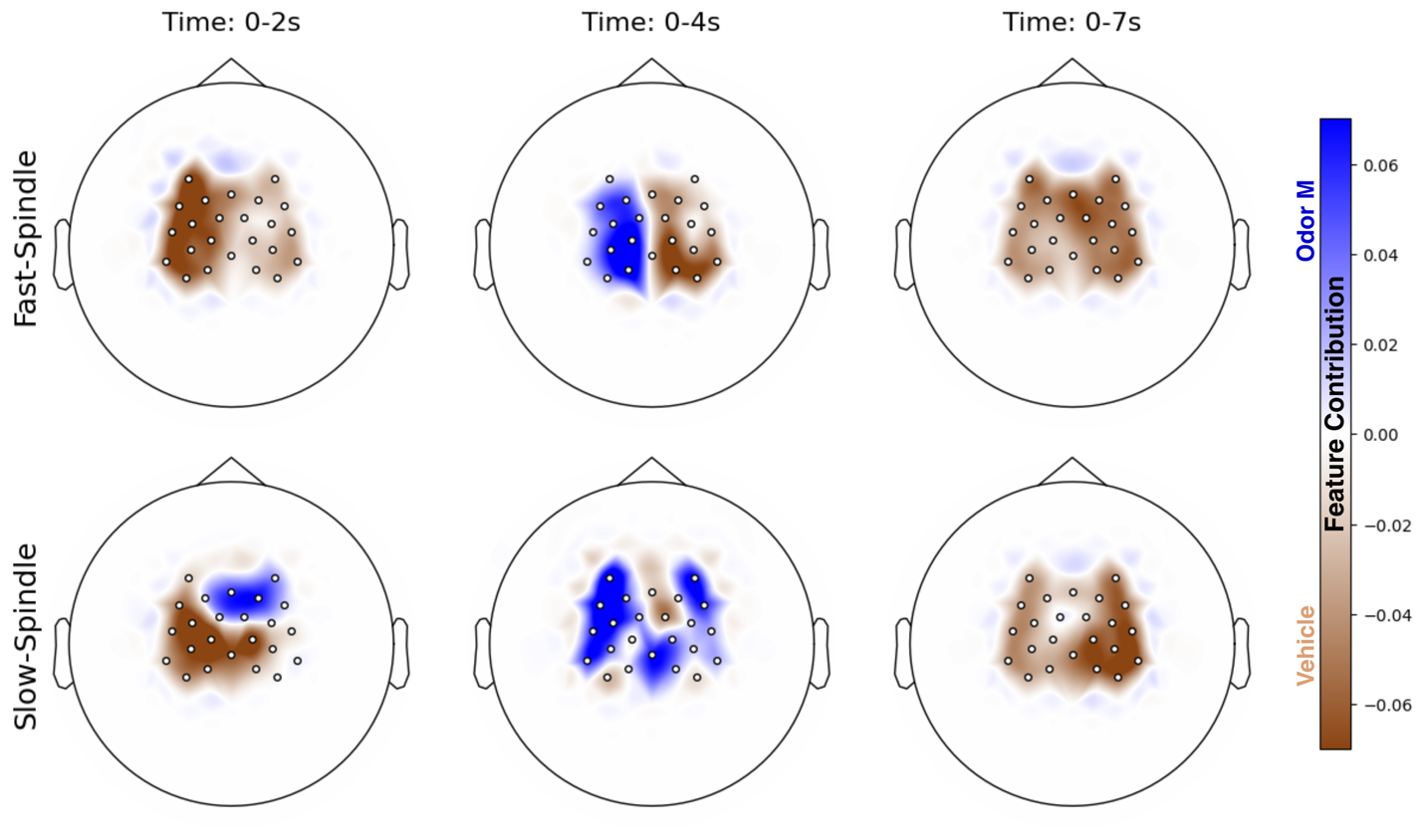


**Supplementary Figure S2**. Selected participants where the mean classification accuracy exceeded the chance level (0.5). Central-channel contributions to odor-based classification in spindle-band EEG (Odor M vs. vehicle) exhibited negative or mixed contributions across frequency bands and time windows. Topographic maps show channel-level feature contributions derived from covariance-based Riemannian decoding for Odor M vs. vehicle, separately for fast- (12.5–16 Hz) and slow-spindle bands (9–12.5 Hz) in the first and second rows, respectively, and across post-stimulus time windows (0–2, 0–4, and 0–7 s). Positive values (red) indicate covariance patterns that support classification toward the Odor M condition, whereas negative values (brown) indicate patterns that support classification toward the vehicle condition.

**1.2 Supplementary Table**

**Supplementary Tables S1-S4**. Participant-level decoding accuracy (mean ± standard deviation) for odor conditions (D and M) relative to vehicle (V) across all and central channel subsets.

## **Table S1. Fast spindle (0–4 s)**

| Participant ID | All (D vs V) | All (M vs V) | Central (D vs V) | Central (M vs V) |
| --- | --- | --- | --- | --- |
| 5 | 0.44 ± 0.12 | 0.55 ± 0.09 | 0.48 ± 0.14 | 0.54 ± 0.12 |
| 9 | 0.67 ± 0.12 | 0.39 ± 0.12 | 0.57 ± 0.14 | 0.59 ± 0.11 |
| 12 | 0.50 ± 0.14 | 0.37 ± 0.12 | 0.49 ± 0.12 | 0.56 ± 0.16 |
| 13 | 0.47 ± 0.19 | 0.50 ± 0.14 | 0.55 ± 0.17 | 0.50 ± 0.12 |
| 14 | 0.63 ± 0.15 | 0.48 ± 0.14 | 0.57 ± 0.17 | 0.52 ± 0.14 |
| 16 | 0.47 ± 0.11 | 0.53 ± 0.11 | 0.41 ± 0.13 | 0.46 ± 0.09 |
| 17 | 0.53 ± 0.12 | 0.54 ± 0.15 | 0.52 ± 0.15 | 0.53 ± 0.11 |
| 20 | 0.44 ± 0.13 | 0.54 ± 0.12 | 0.44 ± 0.10 | 0.47 ± 0.15 |
| 24 | 0.42 ± 0.14 | 0.51 ± 0.13 | 0.59 ± 0.17 | 0.52 ± 0.15 |
| 25 | 0.47 ± 0.09 | 0.43 ± 0.12 | 0.50 ± 0.09 | 0.58 ± 0.16 |
| 26 | 0.67 ± 0.15 | 0.35 ± 0.10 | 0.64 ± 0.14 | 0.37 ± 0.12 |
| 28 | 0.58 ± 0.09 | 0.49 ± 0.11 | 0.55 ± 0.09 | 0.51 ± 0.10 |
| 29 | 0.51 ± 0.17 | 0.52 ± 0.13 | 0.50 ± 0.13 | 0.55 ± 0.14 |
| 30 | 0.50 ± 0.11 | 0.55 ± 0.13 | 0.52 ± 0.14 | 0.43 ± 0.14 |
| 39 | 0.39 ± 0.12 | 0.38 ± 0.17 | 0.42 ± 0.11 | 0.34 ± 0.17 |
| 41 | 0.43 ± 0.09 | 0.42 ± 0.23 | 0.51 ± 0.14 | 0.44 ± 0.19 |
| 44 | 0.60 ± 0.14 | 0.46 ± 0.11 | 0.48 ± 0.10 | 0.43 ± 0.12 |
| 45 | 0.51 ± 0.14 | 0.44 ± 0.16 | 0.45 ± 0.15 | 0.45 ± 0.12 |
| 46 | 0.46 ± 0.13 | 0.44 ± 0.15 | 0.41 ± 0.14 | 0.60 ± 0.13 |
| 47 | 0.43 ± 0.08 | 0.43 ± 0.09 | 0.50 ± 0.10 | 0.43 ± 0.10 |
| 48 | 0.61 ± 0.12 | 0.55 ± 0.12 | 0.68 ± 0.12 | 0.52 ± 0.12 |
| 49 | 0.54 ± 0.11 | 0.59 ± 0.12 | 0.56 ± 0.14 | 0.53 ± 0.12 |
| 51 | 0.48 ± 0.14 | 0.54 ± 0.13 | 0.40 ± 0.12 | 0.46 ± 0.14 |

## **Table S2. Fast spindle (0–7 s)**

| Participant ID | All (D vs V) | All (M vs V) | Central (D vs V) | Central (M vs V) |
| --- | --- | --- | --- | --- |
| 5 | 0.56 ± 0.15 | 0.62 ± 0.12 | 0.43 ± 0.13 | 0.59 ± 0.11 |
| 9 | 0.59 ± 0.13 | 0.54 ± 0.12 | 0.56 ± 0.15 | 0.56 ± 0.10 |
| 12 | 0.56 ± 0.13 | 0.32 ± 0.14 | 0.52 ± 0.11 | 0.48 ± 0.18 |
| 13 | 0.51 ± 0.18 | 0.51 ± 0.12 | 0.56 ± 0.12 | 0.49 ± 0.12 |
| 14 | 0.60 ± 0.19 | 0.39 ± 0.15 | 0.60 ± 0.18 | 0.42 ± 0.15 |
| 16 | 0.48 ± 0.11 | 0.46 ± 0.11 | 0.47 ± 0.13 | 0.47 ± 0.11 |
| 17 | 0.42 ± 0.10 | 0.37 ± 0.13 | 0.54 ± 0.12 | 0.43 ± 0.15 |
| 20 | 0.50 ± 0.11 | 0.44 ± 0.14 | 0.44 ± 0.11 | 0.49 ± 0.12 |
| 24 | 0.37 ± 0.12 | 0.47 ± 0.10 | 0.58 ± 0.15 | 0.49 ± 0.10 |
| 25 | 0.41 ± 0.09 | 0.38 ± 0.11 | 0.48 ± 0.14 | 0.52 ± 0.15 |
| 26 | 0.60 ± 0.11 | 0.37 ± 0.12 | 0.55 ± 0.15 | 0.45 ± 0.11 |
| 28 | 0.57 ± 0.11 | 0.50 ± 0.10 | 0.56 ± 0.08 | 0.48 ± 0.08 |
| 29 | 0.43 ± 0.13 | 0.53 ± 0.15 | 0.43 ± 0.15 | 0.53 ± 0.10 |
| 30 | 0.50 ± 0.12 | 0.42 ± 0.11 | 0.61 ± 0.11 | 0.37 ± 0.12 |
| 39 | 0.42 ± 0.11 | 0.40 ± 0.09 | 0.46 ± 0.11 | 0.38 ± 0.13 |
| 41 | 0.41 ± 0.16 | 0.51 ± 0.16 | 0.49 ± 0.12 | 0.48 ± 0.15 |
| 44 | 0.52 ± 0.14 | 0.43 ± 0.11 | 0.52 ± 0.15 | 0.43 ± 0.11 |
| 45 | 0.51 ± 0.18 | 0.41 ± 0.15 | 0.56 ± 0.11 | 0.42 ± 0.16 |
| 46 | 0.47 ± 0.12 | 0.40 ± 0.13 | 0.49 ± 0.14 | 0.47 ± 0.13 |
| 47 | 0.46 ± 0.08 | 0.48 ± 0.11 | 0.54 ± 0.09 | 0.42 ± 0.10 |
| 48 | 0.45 ± 0.12 | 0.49 ± 0.14 | 0.61 ± 0.12 | 0.51 ± 0.13 |
| 49 | 0.53 ± 0.11 | 0.41 ± 0.11 | 0.57 ± 0.11 | 0.52 ± 0.14 |
| 51 | 0.50 ± 0.12 | 0.51 ± 0.12 | 0.37 ± 0.12 | 0.56 ± 0.15 |

## **Table S3. Slow spindle (0–4 s)**

| Participant ID | All (D vs V) | All (M vs V) | Central (D vs V) | Central (M vs V) |
| --- | --- | --- | --- | --- |
| 5 | 0.57 ± 0.14 | 0.43 ± 0.13 | 0.43 ± 0.12 | 0.59 ± 0.11 |
| 9 | 0.57 ± 0.14 | 0.41 ± 0.13 | 0.59 ± 0.13 | 0.39 ± 0.12 |
| 12 | 0.43 ± 0.14 | 0.39 ± 0.14 | 0.49 ± 0.20 | 0.50 ± 0.19 |
| 13 | 0.41 ± 0.17 | 0.52 ± 0.14 | 0.45 ± 0.16 | 0.50 ± 0.13 |
| 14 | 0.59 ± 0.18 | 0.60 ± 0.18 | 0.65 ± 0.17 | 0.62 ± 0.16 |
| 16 | 0.44 ± 0.13 | 0.46 ± 0.09 | 0.47 ± 0.16 | 0.54 ± 0.13 |
| 17 | 0.50 ± 0.14 | 0.52 ± 0.11 | 0.58 ± 0.16 | 0.50 ± 0.13 |
| 20 | 0.55 ± 0.11 | 0.53 ± 0.10 | 0.53 ± 0.11 | 0.42 ± 0.12 |
| 24 | 0.49 ± 0.14 | 0.38 ± 0.09 | 0.55 ± 0.16 | 0.48 ± 0.14 |
| 25 | 0.44 ± 0.09 | 0.46 ± 0.13 | 0.50 ± 0.13 | 0.52 ± 0.14 |
| 26 | 0.65 ± 0.14 | 0.38 ± 0.11 | 0.65 ± 0.14 | 0.35 ± 0.10 |
| 28 | 0.52 ± 0.10 | 0.47 ± 0.10 | 0.54 ± 0.09 | 0.43 ± 0.09 |
| 29 | 0.60 ± 0.12 | 0.51 ± 0.14 | 0.52 ± 0.17 | 0.54 ± 0.10 |
| 30 | 0.44 ± 0.13 | 0.49 ± 0.16 | 0.49 ± 0.13 | 0.46 ± 0.12 |
| 39 | 0.49 ± 0.14 | 0.43 ± 0.12 | 0.48 ± 0.07 | 0.41 ± 0.12 |
| 41 | 0.51 ± 0.11 | 0.60 ± 0.17 | 0.56 ± 0.15 | 0.52 ± 0.18 |
| 44 | 0.50 ± 0.10 | 0.49 ± 0.11 | 0.51 ± 0.13 | 0.49 ± 0.11 |
| 45 | 0.38 ± 0.13 | 0.43 ± 0.13 | 0.43 ± 0.16 | 0.46 ± 0.11 |
| 46 | 0.52 ± 0.14 | 0.55 ± 0.15 | 0.43 ± 0.16 | 0.55 ± 0.15 |
| 47 | 0.48 ± 0.09 | 0.51 ± 0.07 | 0.43 ± 0.10 | 0.46 ± 0.09 |
| 48 | 0.61 ± 0.14 | 0.52 ± 0.14 | 0.62 ± 0.11 | 0.45 ± 0.14 |
| 49 | 0.48 ± 0.14 | 0.55 ± 0.15 | 0.50 ± 0.13 | 0.43 ± 0.13 |
| 51 | 0.44 ± 0.12 | 0.47 ± 0.14 | 0.36 ± 0.12 | 0.42 ± 0.13 |

## **Table S4. Slow spindle (0–7 s)**

| Participant ID | All (D vs V) | All (M vs V) | Central (D vs V) | Central (M vs V) |
| --- | --- | --- | --- | --- |
| 5 | 0.38 ± 0.14 | 0.45 ± 0.11 | 0.36 ± 0.10 | 0.58 ± 0.12 |
| 9 | 0.64 ± 0.12 | 0.48 ± 0.12 | 0.64 ± 0.13 | 0.46 ± 0.15 |
| 12 | 0.52 ± 0.17 | 0.37 ± 0.14 | 0.52 ± 0.14 | 0.45 ± 0.14 |
| 13 | 0.49 ± 0.15 | 0.45 ± 0.17 | 0.57 ± 0.16 | 0.54 ± 0.15 |
| 14 | 0.54 ± 0.16 | 0.52 ± 0.17 | 0.49 ± 0.14 | 0.46 ± 0.17 |
| 16 | 0.42 ± 0.14 | 0.56 ± 0.09 | 0.48 ± 0.13 | 0.46 ± 0.11 |
| 17 | 0.49 ± 0.14 | 0.43 ± 0.11 | 0.56 ± 0.15 | 0.56 ± 0.17 |
| 20 | 0.51 ± 0.09 | 0.48 ± 0.15 | 0.53 ± 0.12 | 0.39 ± 0.12 |
| 24 | 0.40 ± 0.15 | 0.40 ± 0.12 | 0.60 ± 0.14 | 0.52 ± 0.10 |
| 25 | 0.47 ± 0.09 | 0.48 ± 0.12 | 0.43 ± 0.11 | 0.43 ± 0.12 |
| 26 | 0.60 ± 0.17 | 0.43 ± 0.11 | 0.60 ± 0.13 | 0.50 ± 0.11 |
| 28 | 0.54 ± 0.11 | 0.53 ± 0.08 | 0.58 ± 0.09 | 0.42 ± 0.08 |
| 29 | 0.45 ± 0.12 | 0.46 ± 0.14 | 0.42 ± 0.14 | 0.51 ± 0.11 |
| 30 | 0.49 ± 0.16 | 0.49 ± 0.13 | 0.46 ± 0.15 | 0.49 ± 0.10 |
| 39 | 0.54 ± 0.13 | 0.42 ± 0.11 | 0.57 ± 0.13 | 0.48 ± 0.12 |
| 41 | 0.47 ± 0.15 | 0.54 ± 0.17 | 0.37 ± 0.11 | 0.52 ± 0.14 |
| 44 | 0.50 ± 0.12 | 0.45 ± 0.12 | 0.51 ± 0.13 | 0.55 ± 0.12 |
| 45 | 0.47 ± 0.14 | 0.40 ± 0.12 | 0.57 ± 0.16 | 0.44 ± 0.12 |
| 46 | 0.60 ± 0.11 | 0.56 ± 0.14 | 0.41 ± 0.12 | 0.58 ± 0.21 |
| 47 | 0.50 ± 0.10 | 0.49 ± 0.10 | 0.50 ± 0.11 | 0.42 ± 0.10 |
| 48 | 0.52 ± 0.14 | 0.39 ± 0.13 | 0.55 ± 0.14 | 0.34 ± 0.13 |
| 49 | 0.48 ± 0.10 | 0.41 ± 0.14 | 0.57 ± 0.07 | 0.54 ± 0.14 |
| 51 | 0.42 ± 0.10 | 0.51 ± 0.14 | 0.41 ± 0.12 | 0.49 ± 0.11 |
